# Roseabol A, a New Peptaibol from the Fungus *Clonostachys rosea*

**DOI:** 10.3390/molecules26123594

**Published:** 2021-06-11

**Authors:** Chang-Kwon Kim, Lauren R. H. Krumpe, Emily Smith, Curtis J. Henrich, Isaac Brownell, Karen L. Wendt, Robert H. Cichewicz, Barry R. O’Keefe, Kirk R. Gustafson

**Affiliations:** 1 Molecular Targets Program, Center for Cancer Research, National Cancer Institute, Frederick, MD 21702, USA; kck100639@gmail.com (C.-K.K.); haughl@mail.nih.gov (L.R.H.K.); emily.smith2@nih.gov (E.S.); curtis.henrich2@nih.gov (C.J.H.); okeefeba@mail.nih.gov (B.R.O.); 2 Basic Science Program, Frederick National Laboratory for Cancer Research, Frederick, MD 21702, USA; 3 Dermatology Branch, National Institute of Arthritis and Musculoskeletal and Skin Diseases, Bethesda, MD 20893, USA; isaac.brownell@nih.gov; 4 Natural Products Discovery Group, Department of Chemistry and Biochemistry, University of Oklahoma, Norman, OK 73019, USA; klwendt@ou.edu (K.L.W.); rhcichewicz@ou.edu (R.H.C.); 5 Natural Products Branch, Developmental Therapeutics Program, Division of Cancer Treatment and Diagnosis, National Cancer Institute, Frederick, MD 21701, USA

**Keywords:** peptaibol, *Clonostachys rosea*, Merkel cell carcinoma

## Abstract

A new 11 amino acid linear peptide named roseabol A (**1**) and the known compound 13-oxo-*trans*-9,10-epoxy-11(*E*)-octadecenoic acid (**2**) were isolated from the fungus *Clonostachys rosea*. Combined NMR and MS analysis revealed that roseabol A (**1**) contained amino acid residues characteristic of the peptaibol family of peptides such as isovaline, *α*-aminoisobutyric acid, hydroxyproline, leucinol, and an *N*-terminal isovaleric acid moiety. The amino acid sequence was established by a combination of NMR studies and tandem MS fragmentation analyses, and the absolute configurations of the constituent amino acids of **1** were determined by the advanced Marfey’s method. Compound **2** showed inhibitory activity against Merkel cell carcinoma, a rare and difficult-to-treat type of skin cancer, with an IC_50_ value of 16.5 μM.

## 1. Introduction

Peptaibols are a class of linear polypeptides produced by fungi. Metabolites in this class are 5 to 20 amino acids in length and contain several types of nonproteinogenic amino acid residues such as isovaline, ethylnorvaline, hydroxyproline, and multiple copies of α-aminoisobutyric acid [1,2]. The *N*-terminus is usually acetylated, whereas the *C*-terminal amino acid is amide-linked to a 1,2-amino alcohol. While the majority (>50%) of peptaibols have been isolated from different species of *Trichoderma* [1,2], other fungal genera including *Apiocrea* [3], *Sepedonium* [4], *Clonostachys* [5], and *Paecilomyces* [6] are also known to produce this type of nonribosomal peptide metabolites. Peptaibols are reported to exhibit various biological activities such as antimicrobial [7], antimycoplasmic [8], and inhibition of *β*-amyloid aggregation, which is associated with Alzheimer’s disease [9].

Merkel cell carcinoma (MCC) is a rare but highly aggressive neuroendocrine skin cancer. Although MCC is much less common than other skin cancers such as basal cell carcinoma, squamous cell carcinoma, and melanoma, the incidence of MCC has been increasing rapidly, having quadrupled in the past few decades. This trend is expected to continue, with the projected annual incidence reaching approximately 3250 cases per year in the USA by 2025 [10]. Merkel cell carcinoma tends to grow quickly and spread beyond the skin, making it a difficult cancer to detect and treat soon enough to achieve favorable patient outcomes. The treatment of advanced MCC often utilizes immune checkpoint inhibitors such as avelumab or pembrolizumab [11]. Despite relatively high response rates to these agents, less than half of patients achieve durable benefit; thus, alternative treatments are urgently needed.

**Figure 1:**
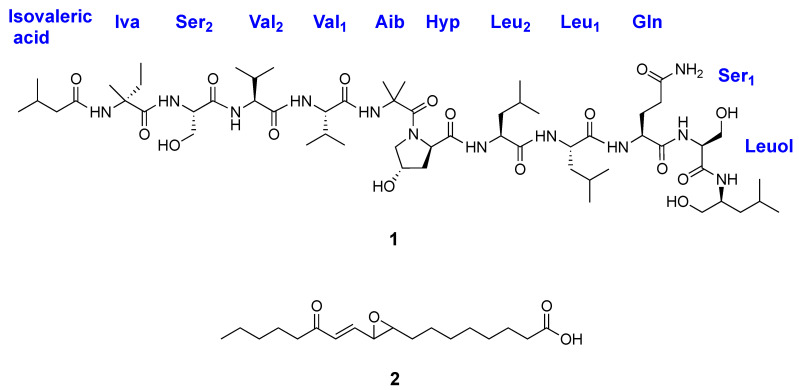
Structures of roseabol A (**1**) and 13-oxo-*trans*-9,10-epoxy-11(*E*)-octadecenoic acid (**2**).

Concurrent with ongoing NCI natural product discovery efforts [12,13], an organic extract of a fungal isolate identified as *Clonostachys rosea* showed significant activity in a cell-based assay for inhibition of Merkel cell carcinoma cell growth and survival. Fractionation of the extract provided a new peptaibol that was named roseabol A (**1**) (Figure 1) and the linoleic acid oxidation product, 13-oxo-*trans*-9,10-epoxy-11(*E*)-octadecenoic acid (**2**) [14]. Based on NMR and MS-MS analyses, it was established that roseabol A (**1**) contained 11 amino acid residues, including those characteristic of peptaibols such as isovaline, *α*-aminoisobutyric acid, hydroxyproline, and leucenol, as well as an *N*-terminal isovaleric acid moiety. Compound **2** was identified by comparison of its spectroscopic data with published data, and it was found to reduce the viability of MCC cells with an IC_50_ value of 16.5 μM. Herein, we report the isolation, structure elucidation, and biological evaluation of compounds **1** and **2**.

## 2. Results and Discussion

### 2.1. Isolation of the Clonostachys rosea Metabolites

The active extract of *C*. *rosea* was subjected to diol column chromatography using a step gradient elution with 100% hexane, CH_2_Cl_2_, EtOAc, acetone, and MeOH. The fraction that eluted with CH_2_Cl_2_ was further separated by C_18_ HPLC using a MeCN–H_2_O gradient to afford 13-oxo-*trans*-9,10-epoxy-11(*E*)-octadecenoic acid (**2**) (0.5 mg). The fraction that eluted with MeOH was subjected to repeated C_18_ HPLC using a MeCN–H_2_O gradient to yield roseabol A (**1**) (1.2 mg).

### 2.2. Structural Characterization of Roseabol A (**1**): Composition and Amino Acid Sequence

The molecular formula of roseabol A (**1**) was deduced to be C_58_H_104_N_12_O_16_ by HRESIMS in conjunction with NMR analyses. The NMR spectra were recorded in DMSO-*d*_6_ as the signals showed well-redispersed resonances and unambiguous 2D-NMR correlations compared to other solvents that were examined (acetone-*d*_6_ and CD_3_OH). The presence of a large number of amide *N*H protons (*δ*_H_ 8.67–6.77) and carbonyl carbons (*δ*_C_ 176.1–169.3) in the ^1^H- and ^13^C-NMR spectra of **1**, respectively, indicated characteristic signals of a peptide (Table 1). Analysis of 2D-NMR (HSQC, COSY, HSQC-TOCSY, HMBC, and ROESY) data led to the assignment of seven common amino acids in **1** consisting of two serine (Ser), one glutamine (Glu), two leucine (Leu), and two valine (Val) residues, as well as one modified amino acid residue hydroxyproline (Hyp). Three nonproteinogenic amino acid residues identified as *α*-aminoisobutyric acid (Aib), isovaline (Iva), and leucinol (Leuol) were also identified. The Aib moiety was readily deduced due to the presence of a quaternary *α*-carbon (*δ*_C_ 56.1) that exhibited HMBC correlations from two singlet methyl groups H_3_-3 (*δ*_H_ 1.44) and H_3_-4 (*δ*_H_ 1.37). NMR resonances of the Iva residue were characterized by the presence of a methyl triplet *δ*_H_ 0.76 (3H, t, *J* = 7.4 Hz) and pronounced shielding of the associated methyl carbon C-4 (*δ*_C_ 7.6) [15], which were indicative of an isolated ethyl group. These data along with HMBC correlations from H_3_-4 (*δ*_H_ 0.76) and H_3_-5 (*δ*_H_ 1.29) to the quaternary carbon C-2 (*δ*_C_ 58.6) supported the assignment of an Iva residue. The *C*-terminal Leuol moiety was established by COSY correlations between N*H* (*δ*_H_ 7.28)/H-1 (*δ*_H_ 3.77), H-1/H_2_-2 (*δ*_H_ 1.28), H-1/H_2_-6 (*δ*_H_ 3.17), and H_2_-2/H-3 (*δ*_H_ 1.45). HMBC correlations from two methyl doublets H_3_-4 (*δ*_H_ 0.79) and H_3_-5 (*δ*_H_ 0.77) to the C-3 carbon (*δ*_C_ 58.6) and from the *α*-methine H-1 to two *β*-methylene carbons C-2 (*δ*_C_ 39.7) and C-6 (*δ*_C_ 63.8) provided further evidence for a Leuol residue. In a similar manner, the *N*-terminal isovaleric acid substituent was confirmed by COSY correlations between H_2_-2/H-3 and H-3/H_3_-4 and H_3_-5, as well as HMBC correlations from H_3_-4 and H_3_-5 to C-3 (*δ*_C_ 25.3) and from H_2_-2 to C-1 (*δ*_C_ 173.1). The *C*-terminus of peptaibols typically consists of an amide-linked amino alcohol such as phenylalaninol, or in some cases leucinol, isoleucinol, valinol, or tryptophanol, while the *N*-terminal residue is usually acetylated [15,16,17,18]. To the best of our knowledge, roseabol A (**1**) is the first example of a fungal peptaibol that is acylated with an *N*-terminal isovaleric acid group.

**Figure 2:**
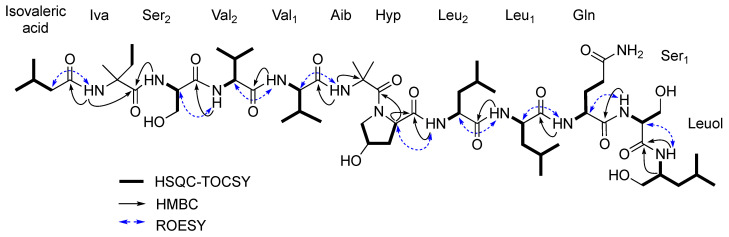
Key HSQC-TOCSY, HMBC, and ROESY correlations for roseabol A (**1**).

The amino acid sequence of roseabol A (**1**) was assembled from a combination of inter-residue ROESY and HMBC correlations (Figure 2). The sequence of isovaleric acid–Iva–Ser_2_–Val_2_–Val_1_–Aib–Hyp–Leu_2_–Leu_1_–Gln–Ser_1_–Leuol was assigned from HMBC correlations between the Leuol NH (*δ*_H_ 7.28)/Ser_1_ C-1 (*δ*_C_ 169.3), Ser_1_ NH (*δ*_H_ 7.62)/Gln C-1 (*δ*_C_ 171.0), Gln NH (*δ*_H_ 7.47)/Leu_1_ C-1 (*δ*_C_ 172.2), Leu_1_ NH (*δ*_H_ 7.35)/Leu_2_ C-1 (*δ*_C_ 172.8), Leu_2_ NH (*δ*_H_ 7.79)/Hyp C-1 (*δ*_C_ 173.3), Hyp H-2 (*δ*_H_ 4.36)/Aib C-1 (*δ*_C_ 173.2), Aib NH (*δ*_H_ 7.90)/Val_1_ C-1 (*δ*_C_ 172.0), Val_1_ NH (*δ*_H_ 7.38)/Val_2_ C-1 (*δ*_C_ 171.5), Val_2_ NH (*δ*_H_ 7.91)/Ser_2_ C-1 (*δ*_C_ 171.4), Ser_2_ NH (*δ*_H_ 8.47)/Iva C-1 (*δ*_C_ 176.1), and Iva NH (*δ*_H_ 8.67)/isovaleric acid C-1 (*δ*_C_ 173.1). The connectivity of the amino acid residues was also confirmed by ROESY data with the following correlations observed: Leuol NH/Ser_1_ H-2 (*δ*_H_ 4.18), Ser_1_ NH/Gln H-2 (*δ*_H_ 4.14), Gln NH/Leu_1_ H-2 (*δ*_H_ 4.24), Leu_1_ NH/Leu_2_ H-2 (*δ*_H_ 4.00), Leu_2_ NH/Hyp H-2 (*δ*_H_ 4.36), Aib NH/Val_1_ H-2 (*δ*_H_ 4.13), Val_1_ NH/Val_2_ H-2 (*δ*_H_ 3.92), Val_2_ NH/Ser_2_ H-2 (*δ*_H_ 4.04), and Iva NH/isovaleric acid H_2_-2 (*δ*_H_ 2.11).

**Figure 3:**
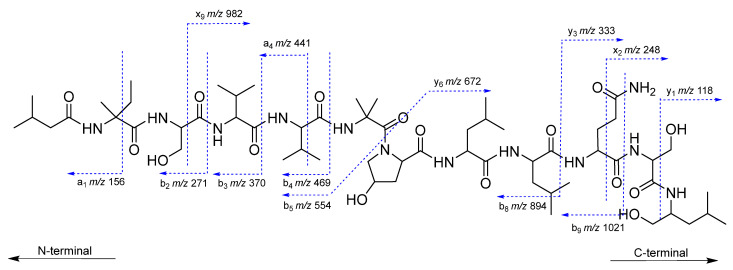
ESI MS/MS fragmentation of roseabol A (**1**).

The sequence of roseabol A (**1**) deduced from the NMR experiments was also supported by ESI-MS/MS collision-induced dissociation analysis. The fragmentation of **1** provided two ions at *m*/*z* 554.3616 (C_27_H_48_N_5_O_7_) and *m*/*z* 672.4314 (C_31_H_58_N_7_O_9_), which were derived from cleavage of the bond between Aib and Hyp to form y_6_ and b_5_ segments [1]. Further analysis of the ESI-MS/MS data revealed a series of a- and b-type fragments (*m*/*z* 1021, 894, 469, 441, 370, 271, and 156) and x- and y-type fragments (*m*/*z* 982, 333, 248, and 118), which were in good agreement with the sequential loss of the assigned amino acid residues of **1** from the *C*-terminus and *N*-terminus, respectively (Figure 3 and Figure S10). Thus, the planar structure of roseabol A (**1**) was elucidated.

### 2.3. Assignment of the Relative and Absolute Configuration of Roeseabol A (**1**)

**Figure 4:**
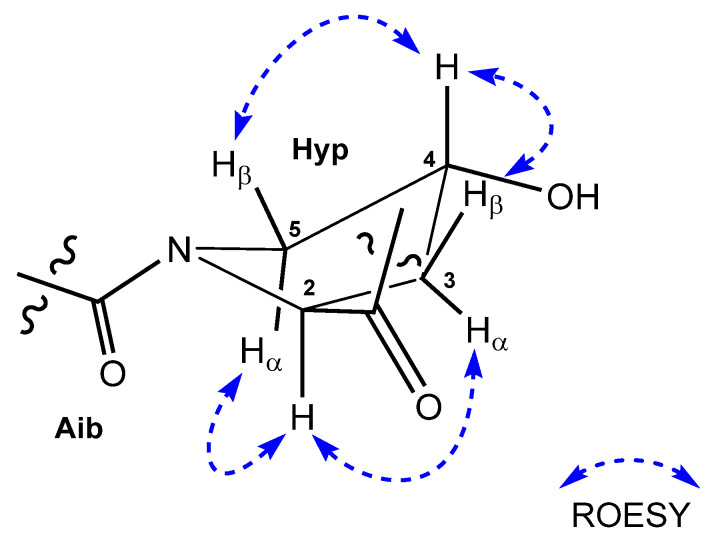
ROESY correlations of *trans*-4-OH-proline.

The relative configuration of the Hyp unit was determined from ROESY correlations between the *α*-proton H-2 (*δ*_H_ 4.36) and both H-3*α* (*δ*_H_ 2.13) and H-5*α* (*δ*_H_ 3.64), whereas the hydroxy methine proton H-4 (*δ*_H_ 4.25) had ROESY correlations to H-3*β* (*δ*_H_ 1.62) and H-5*β* (*δ*_H_ 3.24). Thus, the relative stereochemistry was assigned to be *trans* with the configurations of C-2 as *S* * and C-4 as *R* * (Figure 4).

The absolute configurations of the amino acid constituents of roseabol A (**1**) were determined by acid hydrolysis and application of the advanced Marfey’s method [19,20]. The resulting amino acids were derivatized with 1-fluoro-2, 4-dinitrophenyl-5-L-leucinamide (FDLA) and analyzed by LC-MS using ion-selective monitoring. Comparison of the retention times of the L- and D-DLA derivatives generated from the hydrolysate of **1** with similar derivatives of appropriate amino acid standards allowed assignment of the absolute configuration. When the molecular ion at *m*/*z* = 412 was monitored in the positive ion mode, the DLA-containing products from three amino acid moieties Val, Leuol, and Iva were detected. However, these products were well resolved as the L-DLA derivatives of L- and D-Val eluted at 24.9 and 29.1 min, L- and D-Leuol at 27.2 and 31.5 min, and L- and D-Iva at 26.1 and 27.7 min, respectively (Figure S11). The results from **1** indicated that the two Val residues and Leuol had L-configurations while Iva had a D-configuration. In a similar manner, the absolute configurations of L-Ser, L-Gln, L-Leu, and *trans*-D-Hyp in **1** were established (Figures S12–S14). Thus, the absolute configurations at all 11 stereogenic centers in roseabol A (**1**) were unambiguously established.

### 2.4. Characterization of 13-Oxo-Trans-9,10-Epoxy-11(E)-Octadecenoic Acid (**2**)

The linoleic acid oxidation product 13-oxo-*trans*-9,10-epoxy-11(*E*)-octadecenoic acid (**2**) was identified by analysis of its spectroscopic (NMR) and spectrometric (HRMS) data and through comparisons with literature data [14,21]. The epoxide protons of **2** were assigned a *trans*-configuration based on the shielding effect observed for their NMR signals relative to the corresponding *cis*-epoxide product.

### 2.5. Assessment of Activity Against Merkel Cell Carcinoma

Compounds **1** and **2** were tested for cytotoxic activity against two Merkel cell carcinoma cell lines; MCC26, which is free of the Merkel cell polyomavirus (MCPyV), and MKL-1, which is positive for the virus. Approximately 80% of clinical Merkel cell carcinomas show clonal integration of MCPyV in their DNA [22]. The keratinocyte cell line HaCaT was also included in the assay system as a noncancerous control cell line. Compound **2** showed cytotoxic activity towards MKL-1 cells (IC_50_ of 16.5 μM) and MCC26 cells (IC_50_ = 25.6 μM) but was not toxic to the HaCaT control cells (Figure S16). The new metabolite roseabol A (**1**), while expanding and diversifying the known chemical space of the peptaibol family of peptides, was inactive against all three cell lines. The inhibitory activity that **2** exhibits toward the MCC cell lines suggests it may have some utility as a molecular probe to help define targetable processes important for the initiation, maintenance, and progression of Merkel cell carcinoma.

## 3. Materials and Methods

### 3.1. General Experimental Procedures

Optical rotation measurements were made on a Rudolph research analytical AUTOPOL IV automatic polarimeter (Rudolph Research Analytical, Hackettstown, NJ, USA), IR spectra were recorded with a Bruker ALPHA II FT-IR spectrometer (Bruker, Billerica, MA, USA), and UV spectra were measured with a Thermo Scientific Nanodrop 2000C spectrophotometer (Thermo Fisher Scientific, Waltham, MA, USA). The ECD spectrum was obtained on a JASCO J-1500 circular dichroism spectrometer (Jasco, Easton, MD, USA). High-performance liquid chromatography (HPLC) was performed using a Varian ProStar 215 solvent delivery module equipped with a Varian ProStar 320 UV-Vis detector (Agilent Technologies, Santa Clara, CA, USA), operating under Star 6.41 chromatography workstation software (Agilent Technologies, Santa Clara, CA, USA). NMR spectra were obtained with a Bruker Avance III NMR spectrometer (Bruker, Billerica, MA, USA) equipped with a 3 mm cryogenic probe and operating at 600 MHz for ^1^H and 150 MHz for ^13^C. Spectra were calibrated to residual solvent signals at *δ*_H_ 2.50 and *δ*_C_ 39.5 in DMSO-*d*_6_. All 2D-NMR experiments were acquired with nonuniform sampling (NUS) set to 50% or 25%. HRESIMS data were acquired on an Agilent Technologies 6530 Accurate-Mass Q-TOF LC/MS instrument (Agilent Technologies, Santa Clara, CA, USA).

### 3.2. Fungal Isolation, Culture, and Extraction

The *Clonostachys rosea* isolate (MI4762 TV8-1) was obtained from a soil sample collected from Macomb, MI, USA, and submitted to the Citizen Science Soil Collection Program at the University of Oklahoma. Copies of the fungus are permanently maintained under cryogenic storage conditions in the University of Oklahoma Citizen Science Soil Collection Program Repository. The region spanning ITS1–5.8S–ITS2 of the genomic DNA was sequenced (GenBank accession number MW466525). Based on comparisons of the resulting sequence to sequences deposited in GenBank, the fungus exhibited a 100% identity match with multiple *Clonstachys rosea* isolates and was identified as a member of this species. The fungus was grown on Cheerios breakfast cereal supplemented with 0.3% sucrose and 0.005% chloramphenicol in three large mycobags (Unicorn Bags, Plano, TX, USA) for four weeks at room temperature. The fungal biomass was extracted overnight in ethyl acetate. The resulting organic extract was twice subjected to partitioning with water (1:1, vol:vol). The ethyl acetate layer was retained and the organic solvent was evaporated in vacuo, yielding approximately 18 g of deep red organic-soluble material.

### 3.3. Compound Isolation

A 6.4 g aliquot of the fungal extract was subjected to diol reversed-phase flash chromatography using step gradient elution with 100% hexane (fraction A, 368 mg), 100% CH_2_Cl_2_ (fraction B, 504 mg), 100% EtOAc (fraction C, 2.2 g), 100% acetone (fraction D, 2.1 g), and 100% MeOH (fraction E, 372.2 mg). The active fraction B was separated by preparative reversed-phase HPLC using a Dynamax C18 column (Agilent Technologies, Santa Clara, CA, USA), 21.4 mm × 250 mm, 9.0 mL/min, CH_3_CN–H_2_O gradient (20:80–100:0), detection at 254 nm, yielding 11 peaks rich in secondary metabolites. Purification of subfraction 7 was accomplished by semipreparative HPLC on a Luna C18 column (Phenomenex, Torrance, CA, USA), 10 mm × 250 mm, 3.0 mL/min, MeCN–H_2_O gradient (40:60–100:0), detection at 254 nm, and further purified by analytical HPLC using a Luna C18 column (Phenomenex, Torrance, CA, USA), 4.6 mm × 250 mm, 0.9 mL/min, MeCN–H_2_O gradient (50:50–100:0), detection at 254 nm, to yield compound **2** (0.5 mg) as an amorphous solid. The active fraction E was separated by preparative reversed-phase HPLC on a Dynamax C18 column (Agilent Technologies, Santa Clara, CA, USA), 21.4 mm × 250 mm, 9 mL/min, MeCN–H_2_O gradient (40:60–100:0), detection at 220 nm, yielding 9 peaks rich in secondary metabolites. Further purification of subfraction 9 by analytical HPLC using a Luna C18 column (Phenomenex, Torrance, CA, USA), 4.6 mm × 250 mm, 0.9 mL/min, MeCN–H_2_O gradient (42:58–100:0), detection at 220 nm, afforded 1.2 mg of roseabol A (**1**) as an amorphous solid.

*Roseabol A* (**1**): white, amorphous solid; [α]25D +7.7 (*c* 0.5, MeOH); UV (MeOH) λ_max_ (log *ε*) 195 (3.57) ; CD (*c* 3.3 × 10^−4^ M, MeOH) λ_max_ (Dε) 194 (+30.79), 206 (−40.01), 224 (−13.69) nm; IR (film) *ν*_max_ 3286, 2935, 1650, 1540, 1440, 1384, 1201, 1058 cm^−1^; ^1^H- and ^13^C-NMR, Table 1; HRESIMS *m*/*z* 1225.7774 [M + H]^+^ (calcd for C_58_H_105_N_12_O_16_, 1225.7770).

### 3.4. Acid Hydrolysis of Roseabol A (**1**) and LC-MS Analysis of Marfey’s Derivatives

Two 0.1 mg aliquots of **1** were individually dissolved in degassed 6 N HCl (0.6 mL) and heated in sealed glass vials at 110 °C for 17 h. The hydrolysates were evaporated to dryness and dissolved in H_2_O (50 *μ*L); to this solution was added 20 *μ*L of 1 N NaHCO_3_ and 100 *μ*L of a 1% solution in acetone of either 1-fluoro-2,4-dinitrophenyl-5-L-leucinamide (L-FDLA) or a racemic mixture of D/L-FDLA [19,20]. The reaction mixtures were heated to 40 °C for 40 min and then cooled to room temperature, neutralized with 2 N HCl (20 *μ*L), and evaporated to dryness. The residue was dissolved in CH_3_CN/H_2_O (1:1) and then analyzed by LC-MS on a Poroshell 120 EC-C18 column (Agilent Technologies, Santa Clara, CA, USA), 4.6 × 150 mm, 1.0 mL/min, CH_3_CN–H_2_O gradient (5:95–100:0), containing 0.1% formic acid in 60 min. An Agilent 6130 Quadrupole mass spectrometer (Agilent Technologies, Santa Clara, CA, USA) was used for ESIMS detection (positive and negative ion mode). FDLA derivatives were detected by absorption at 340 nm, and assignment was secured by ion-selective monitoring. The retention times (*t*_R_) of the D/L-DLA mixtures (with the L-DLA *t*_R_ underlined) were as follows:

*Roseabol A* (**1**): L-Val (24.9), D-Val (29.1), *m*/*z* 412 [M + H]^+^; L-Iva (26.1), D-Iva (27.7), *m*/*z* 412 [M + H]^+^; L-Leuol (27.2), D-Leuol (31.5), *m*/*z* 412 [M + H]^+^; *trans*-L-Hyp (18.8), *trans*-D-Hyp (19.1), *m*/*z* 426 [M + H]^+^; L-Leu (26.7), D-Leu (31.3), *m*/*z* 426 [M + H]^+^; L-Ser (20.8), D-Ser (21.3), *m*/*z* 400 [M + H]^+^; L-Gln (21.8), D-Gln (22.7), *m*/*z* 440 [M − H]^−^.

### 3.5. Merkel Cell Carcinoma Assay

The purified compounds were assessed for growth inhibition/cytotoxicity against two Merkel cell carcinoma cell lines, MCC26 (MCPyV−) and MKL-1 (MCPyV+), as well as an immortalized human keratinocyte cell line (HaCaT). Briefly, cells were plated in 384-well clear tissue culture plates with 2500 cells/well (MCC26, HaCaT) or 15,000 cells/well (MKL1) in DME/10% FBS (MCC26, MKL1) or RPMI/10% FBS (HaCaT). The plated cells were allowed to grow overnight, followed by addition of the test compound. DMSO solutions of the compounds were diluted in growth medium prior to addition and assessed in a 10-point (1:2) dilution series, top concentration 40 µM. After 3 days, relative cell numbers were assessed using the XTT metabolic growth assay [23]. The resulting signal was normalized to that of the vehicle control (DMSO) for each cell line. IC_50_ values were estimated from dose–response curves using 4-parameter logistic analysis (SigmaPlot, San Jose, CA, USA).

## 4. Conclusions

A new 11-residue peptide named roseabol A (**1**) was isolated and characterized from the fungus *Clonostachys rosea.* Studies of secondary metabolite production by various *Clonostachys* isolates have only been sparsely reported in the chemical literature. There is one prior report of a peptaibol [5], as well as two *N*-methylated cyclic peptides [24] and a series of polyketide derivatives [25] that have been described from this fungal genus. Roseabol A (**1**) is only the second member of the peptaibol family of peptides to be discovered from a *Clonostachys* isolate. The oxidized linoleic acid derivative 13-oxo-*trans*-9,10-epoxy-11(*E*)-octadecenoic acid (**2**) was also obtained from the *C. rosea* extract. Compound **2** is an epoxyketooctadecenoic acid derivative that is known to stimulate corticosterone production [26] and activate the antioxidant response element [27]. It has also been the focus of recent synthetic efforts [21]. Compound **2** showed selective cytotoxic activity towards the Merkel cell carcinoma cell line MKL-1, which is positive for the Merkel cell polyomavirus (MCPyV+), and was less effective against the MCPyV− MCC26 cell line. Roseabol A (**1**) was inactive against both Merkel cell carcinoma cell lines. The differential response seen between the virus-positive and virus-negative cell lines suggests that **2**, or a related structural analog, could have value as a biological probe to investigate virus-associated aspects of Merkel cell carcinoma.

## Figures and Tables

**Table 1:** ^1^H-NMR (600 MHz) and ^13^C-NMR Data (150 MHz) of roseabol A (**1**) in DMSO-*d*_6._

Position	*δ*_C_, Type	*δ*_H_, (*J* in Hz)	Position	*δ*_C_, Type	*δ*_H_, (*J* in Hz)
	**Leuol**		3	37.0, CH_2_ (α)	2.13, m
1	48.8, CH	3.77, m		(β)	1.62, m
2	39.7, CH_2_	1.56, m	4	69.0, CH	4.25, br s
		1.28, m	5	56.4, CH_2_ (α)	3.64, br d (11.8)
3	23.4, CH	1.45, m		(β)	3.24, br d (11.8)
4	21.9, CH_3_	0.79, d (6.4)		**Aib**	
5	20.8, CH_3_	0.77, d (6.4)	1	173.2, C	
6	63.8, CH_2_	3.28, m	2	56.1, C	
		3.17, m	3	23.1, CH_3_	1.44, s
NH		7.28, d (8.9)	4	25.5, CH_3_	1.37, s
	**Ser_1_**		NH		7.90, s
1	169.3, C			**Val_1_**	
2	55.5, CH	4.18, dd (9.8, 6.5)	1	172.0, C	
3	61.6, CH_2_	3.58, br d (5.6)	2	58.3, CH	4.13, m
NH		7.62, d (7.9)	3	29.4, CH	2.15, m
	**Gln**		4	17.8, CH_3_	0.87, d (6.7)
1	171.0, C		5	19.2, CH_3_	0.94, d (6.7)
2	52.8, CH	4.14, m	NH		7.38, d (7.8)
3	27.5, CH_2_	1.90, m		**Val_2_**	
		1.76, m	1	171.5, C	
4	31.5, CH_2_	2.13, m	2	60.4, CH	3.92, t (6.5)
		2.07, m	3	29.1, CH	2.14, m
5	173.7, C		4	18.8, CH_3_	0.86, d (6.5)
2-NH		7.47, d (6.8)	5	19.0, CH_3_	0.94, d (6.5)
5-NH_2_		6.77, s	NH		7.91, d (7.8)
		7.24, s		**Ser_2_**	
	**Leu_1_**		1	171.4, C	
1	172.2, C		2	57.8, CH	4.04, m
2	51.2, CH	4.24, m	3	60.7, CH_2_	3.70, m
3	39.2, CH_2_	1.77, m			3.66, m
		1.28, m	NH		8.47, m
4	24.4, CH	1.60, m		**Iva**	
5	22.3, CH_3_	0.87, d (6.6)	1	176.1, C	
6	22.2, CH_3_	0.77, d (6.6)	2	58.6, C	
NH		7.35, d (7.8)	3	27.1, CH_2_	1.92, m
	**Leu_2_**				1.69, m
1	172.8, C		4	7.6, CH_3_	0.76, t (7.4)
2	52.5, CH	4.00, m	5	22.1, CH_3_	1.29, s
3	38.8, CH_2_	1.86, m	NH		8.67, s
		1.54, m		**Isovaleric acid**	
4	24.0, CH	1.58, m	1	173.1, C	
5	22.8, CH_3_	0.87, d (6.6)	2	44.4, CH_2_	2.11, m
6	22.3, CH_3_	0.77, d (6.6)			2.07, m
NH		7.79, d (7.8)	3	25.3, CH	1.99, m
	**Hyp**		4	22.4, CH_3_	0.79, d (6.7)
1	173.3, C		5	24.1, CH_3_	0.84, d (6.7)
2	61.3, CH	4.36, t (8.6)			

## Supplementary Materials

The following are available online. Figures S1–S8: ^1^H,^13^C-NMR, edited HSQC, ^1^H-^1^H COSY, HSQC-TOCSY, HMBC, ROESY NMR spectra of compound **l**, Figures S9 and S10: HR-ESI MS data and MS/MS fragmentations for **1**, Figures S11–S14: Advanced Marfey’s analysis for **1**, Figure S15: ECD spectrum of **1**, Figure S16: Merkel cell carcinoma cytotoxic activity of compound **2**.

## Sample Availability

Samples of the compounds **1** and **2** are available from the authors.
