# Supplementary material for: Roseabol A, a New Peptaibol from the Fungus Clonostachys rosea
Source: Molecules. 2021 Jun 11;26(12):3594. doi: 10.3390/molecules26123594 (PMC8231123; doi:10.3390/molecules26123594)
Supplement: Supplementary file 1 [file molecules-26-03594-s001.zip › molecules-1245868-supplementary.pdf]

Supplementary data

Roseabol A, a New Peptaibol from the Fungus *Clonostachys rosea*

Chang-Kwon Kim,[1] Lauren R. H. Krumpe,[1,2] Emily Smith,[1,2] Curtis J. Henrich,[1,2] Isaac Brownell,[3] Karen L. Wendt,[4] Robert H. Cichewicz,[4] Barry R. O'Keefe,[1,5] and Kirk R. Gustafson[1,*]

1 Molecular Targets Program, Center for Cancer Research, National Cancer Institute, Frederick, Maryland 21702-1201, USA; chang-kwon.kim@nih (C.-K.K.); haughl@.mail.nih.gov (L.R.H.K.); emily.smith2@nih.gov (E.S.); curtis.henrich2@nih.gov C.J.H.); okeefeba@mail.nih.gov (B.R.O.)

2 Basic Science Program, Frederick National Laboratory for Cancer Research, Frederick, Maryland, 21702-1201, USA

3 Dermatology Branch, National Institute of Arthritis and Musculoskeletal and Skin Diseases, Bethesda, Maryland, 20893, USA; isaac.brownell@nih.gov

4 Natural Products Discovery Group, Department of Chemistry and Biochemistry, University of Oklahoma, Norman, OK, 73019-5251, USA; klwendt@ou.edu (K.L.W.); rhcichewicz@ou.edu (R.H.C.)

5 Natural Products Branch, Developmental Therapeutics Program, Division of Cancer Treatment and Diagnosis, National Cancer Institute, Frederick, Maryland 21701-1201, USA

Contents

Figure S1. ^{1}H NMR (600 MHz, DMSO-d_6) spectrum of **1** S3
Figure S2. ^{1}H NMR (600 MHz, Acetone-d_5) spectrum of **1** S4
Figure S3. ^{13}C NMR (150 MHz, DMSO-d_6) spectrum of **1** S5
Figure S4. Edited HSQC NMR (600 MHz, DMSO-d_6) spectrum of **1** S6
Figure S5. COSY NMR (600 MHz, DMSO-d_6) spectrum of **1** S7
Figure S6. HSQC-TOCSY NMR (600 MHz, DMSO-d_6) spectrum of **1** S8
Figure S7. HMBC NMR (600 MHz, DMSO-d_6) spectrum of **1** S9
Figure S8. ROESY NMR (600 MHz, DMSO-d_6) spectrum of **1** S10
Figure S9. HRESI-MS data for **1** S11
Figure S10. HRESI-MS/MS fragmentations for **1** S12
Figure S11. Advanced Marfey's analysis for Iva, Val, and Leuol residues of **1** S13
Figure S12. Advanced Marfey's analysis for *trans*-Hyp and Leu residues of **1** S14
Figure S13. Advanced Marfey's analysis for Ser residue of **1** S15
Figure S14. Advanced Marfey's analysis for Gln residue of **1** S16
Figure S15. ECD spectrum of **1** S17
Figure S16. Merkel cell carcinoma cytotoxic activity of compound **2** S18

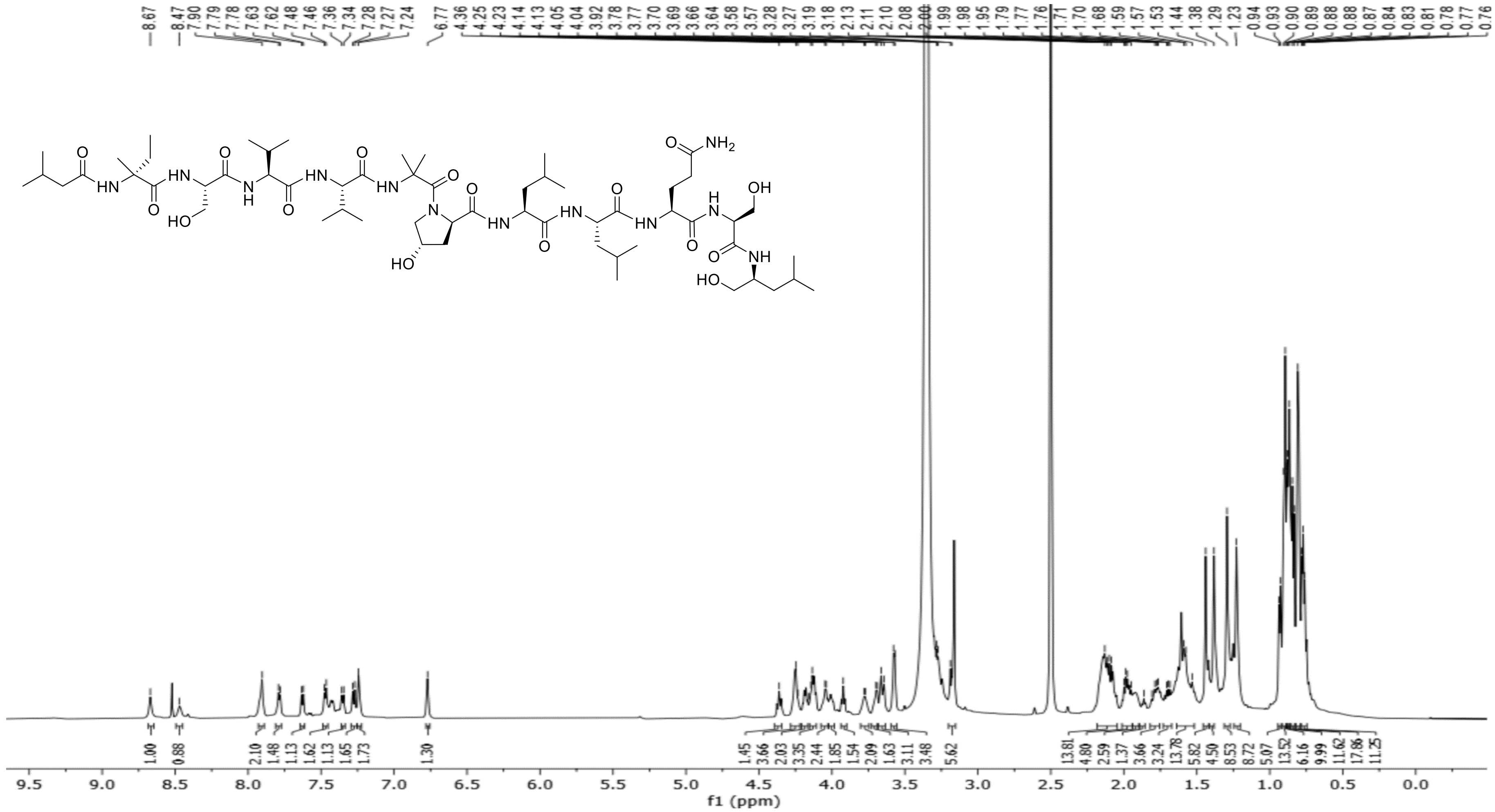

Figure S1. ^{1}H NMR (600 MHz, DMSO-d_6) spectrum of **1**

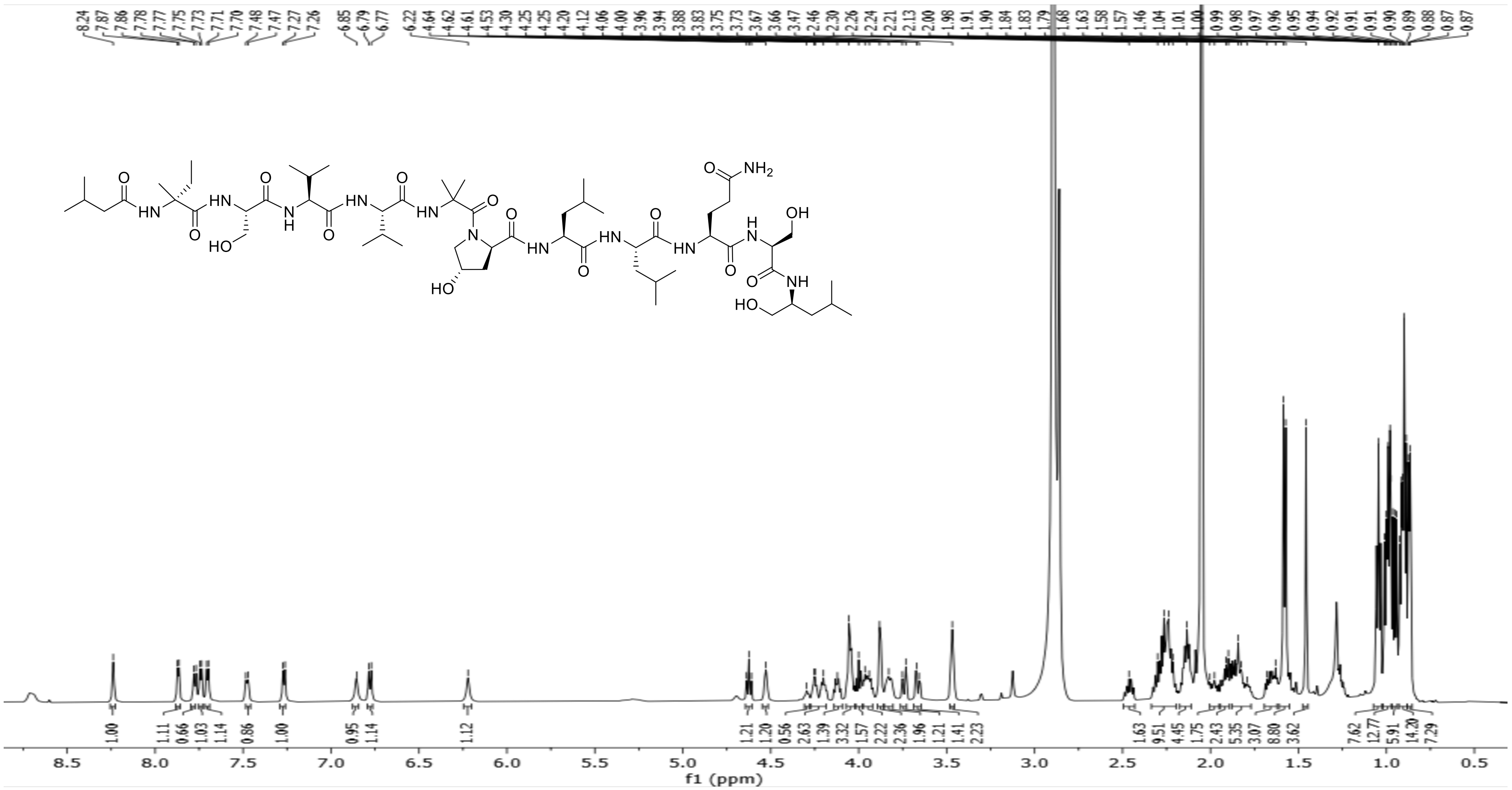

Figure S2. ^{1}H NMR (600 MHz, Acetone-d_6) spectrum of **1**

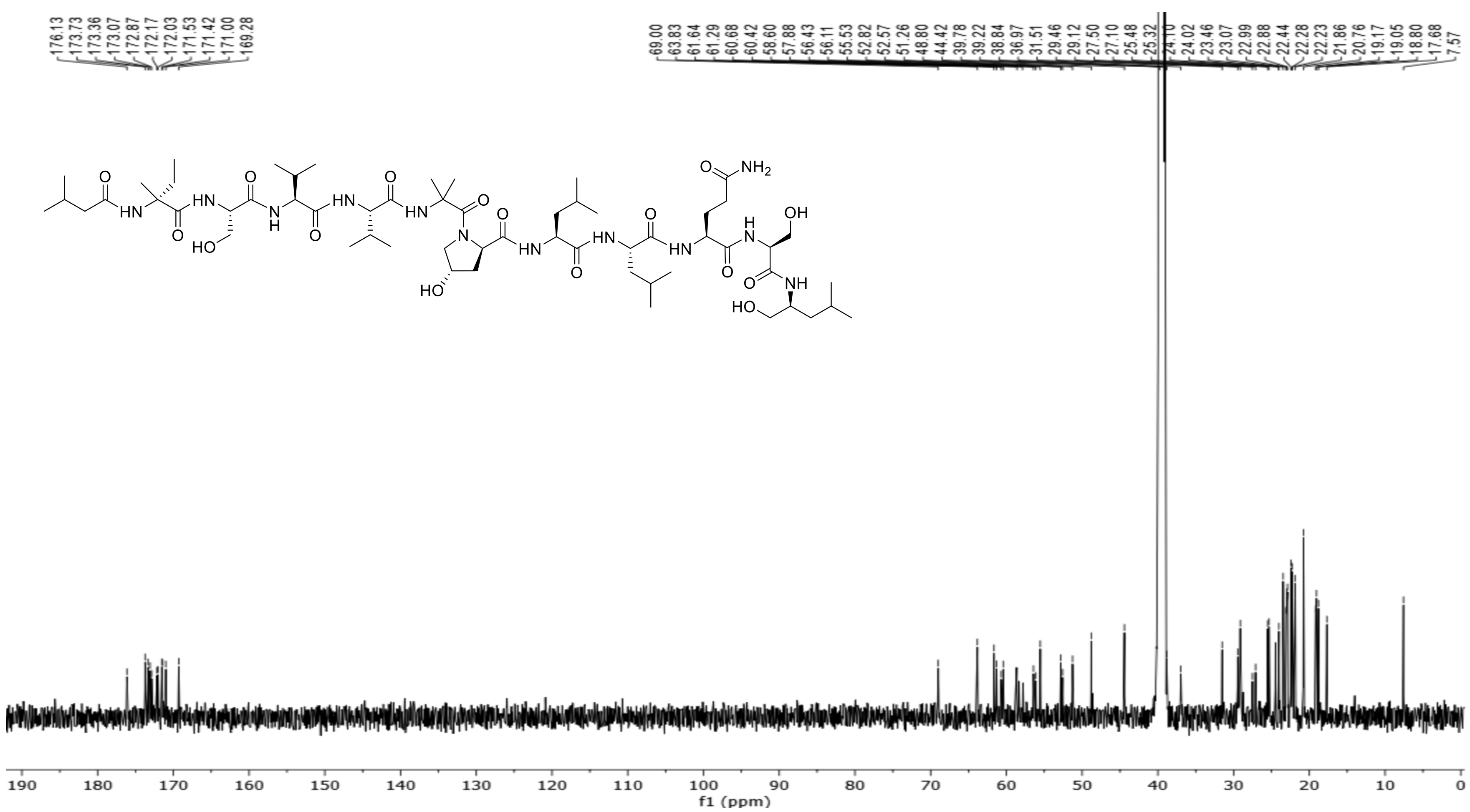

Figure S3. ^{13}C NMR (150 MHz, DMSO-d_6) spectrum of **1**

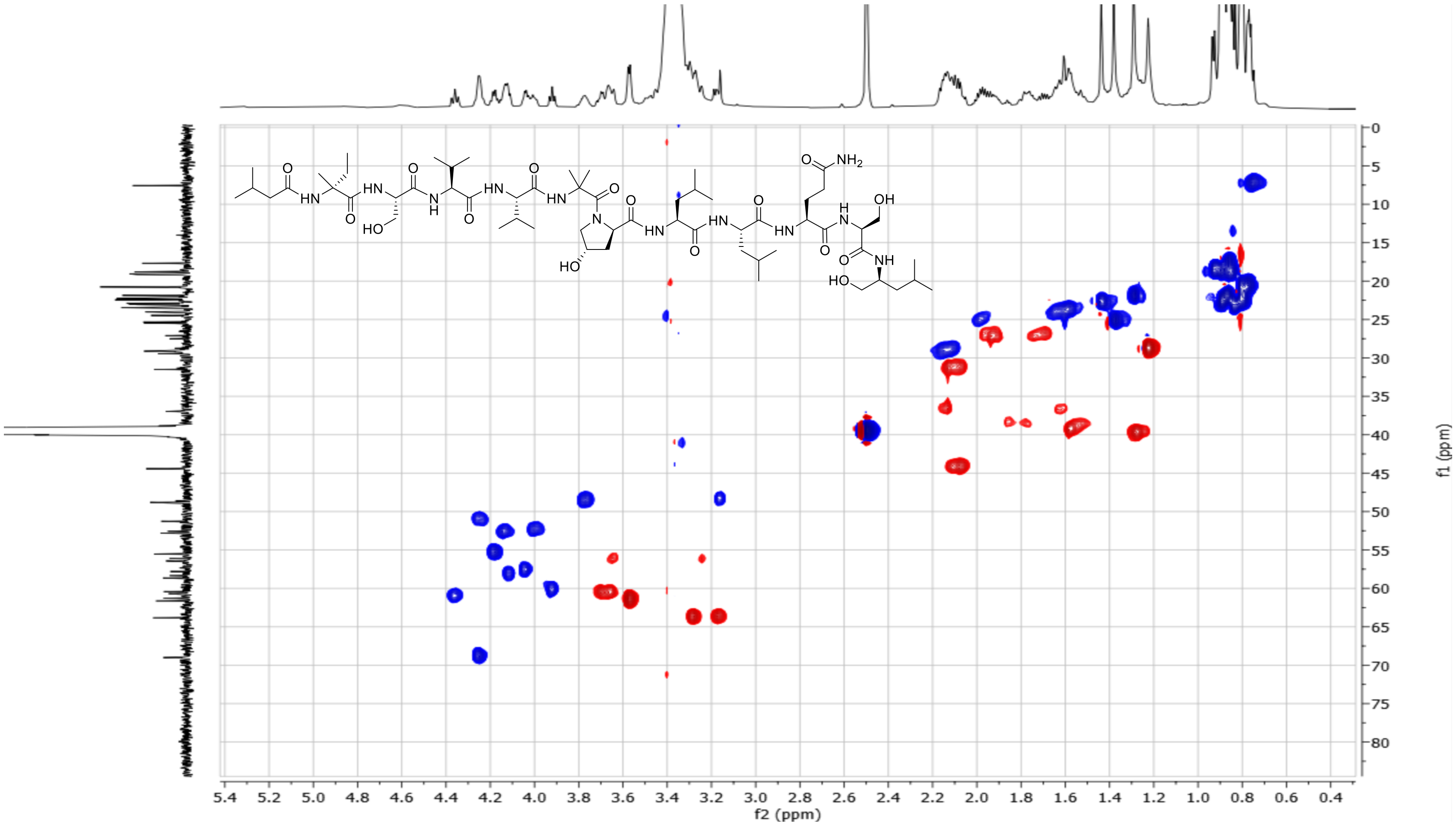

Figure S4. Edited HSQC NMR (600 MHz, DMSO-d_6) spectrum of **1**

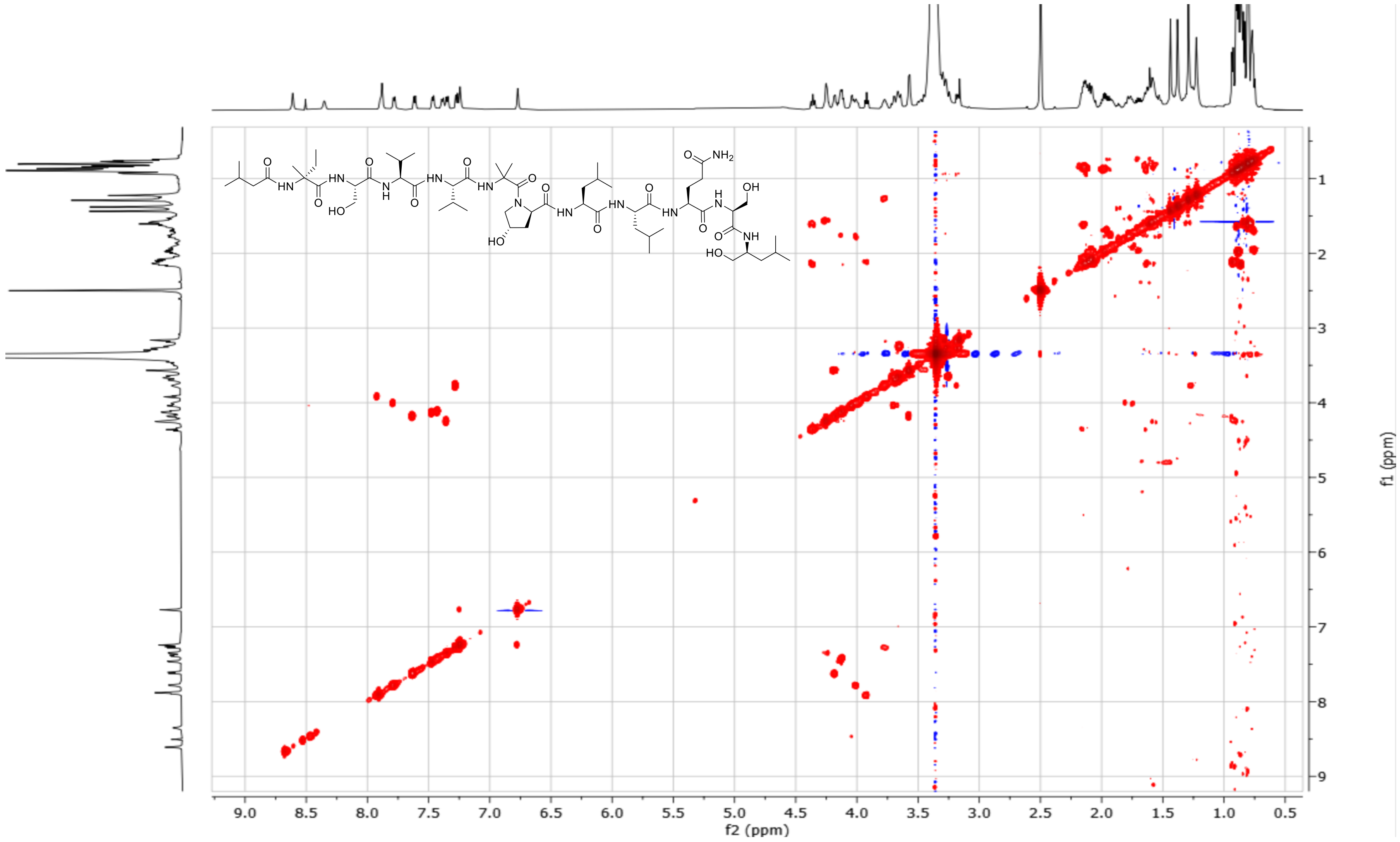

Figure S5. COSY NMR (600 MHz, DMSO-d_6) spectrum of **1**

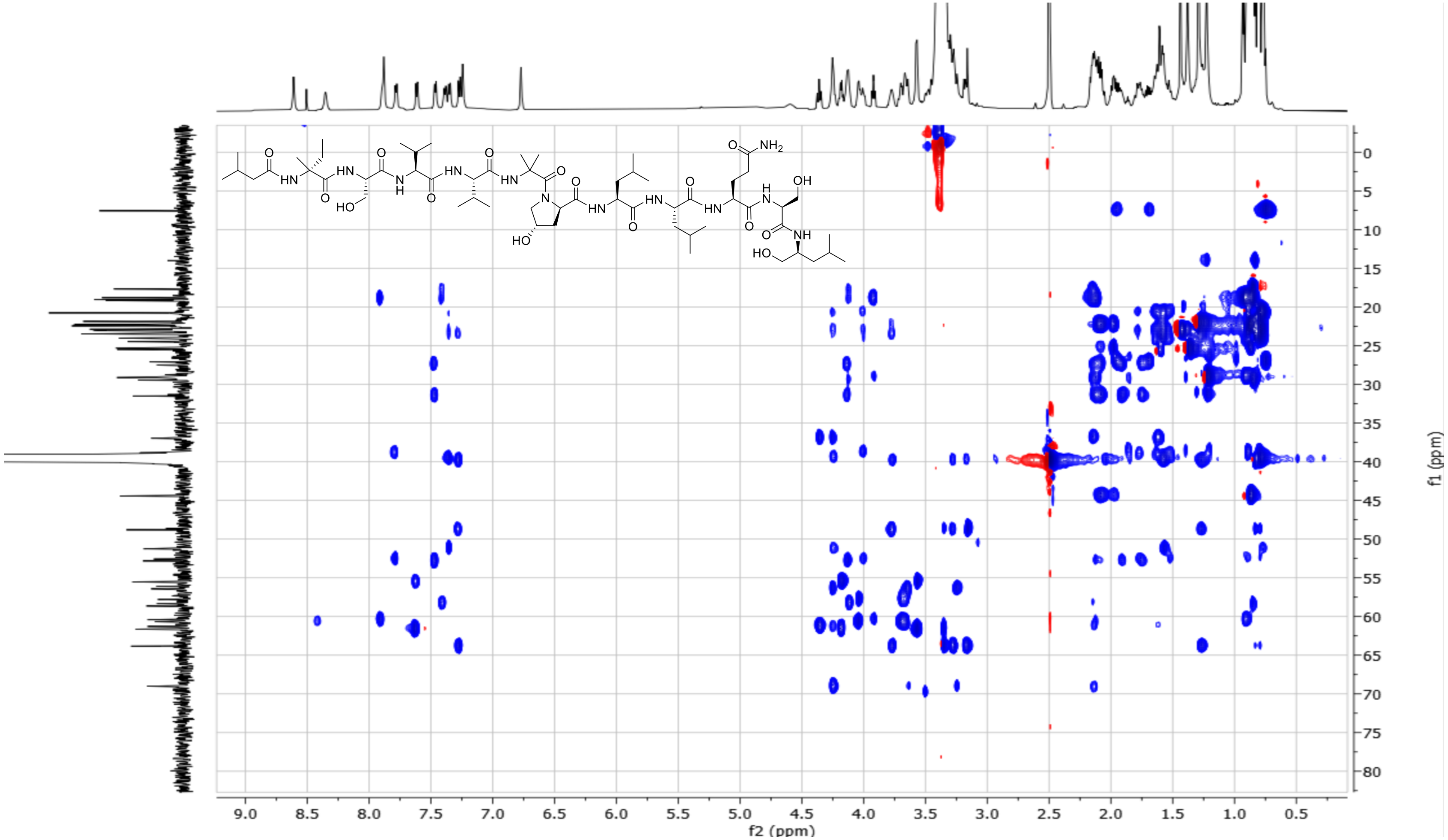

Figure S6. HSQC-TOCSY NMR (600 MHz, DMSO-d_6) spectrum of **1**

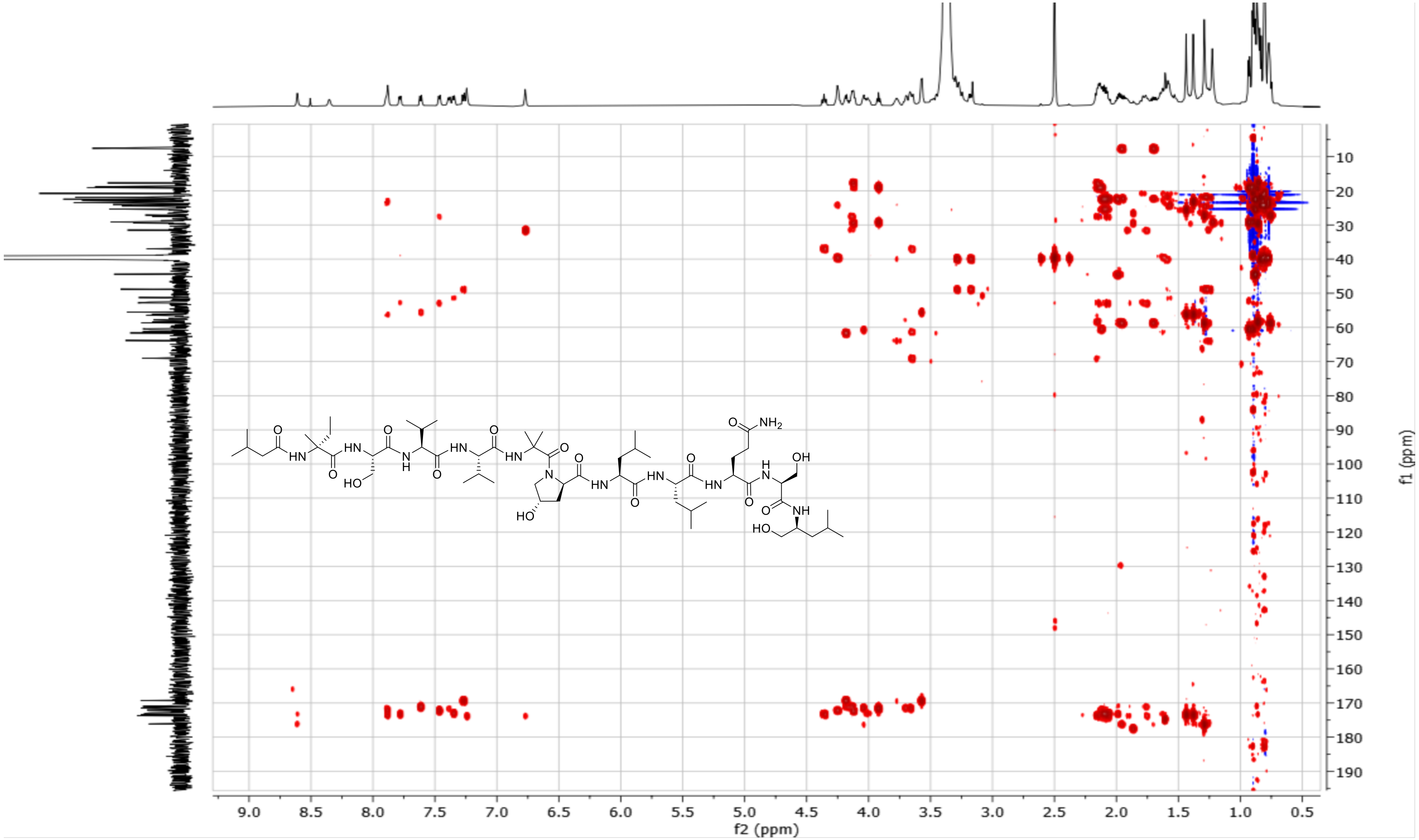

Figure S7. HMBC NMR (600 MHz, DMSO-d_6) spectrum of **1**

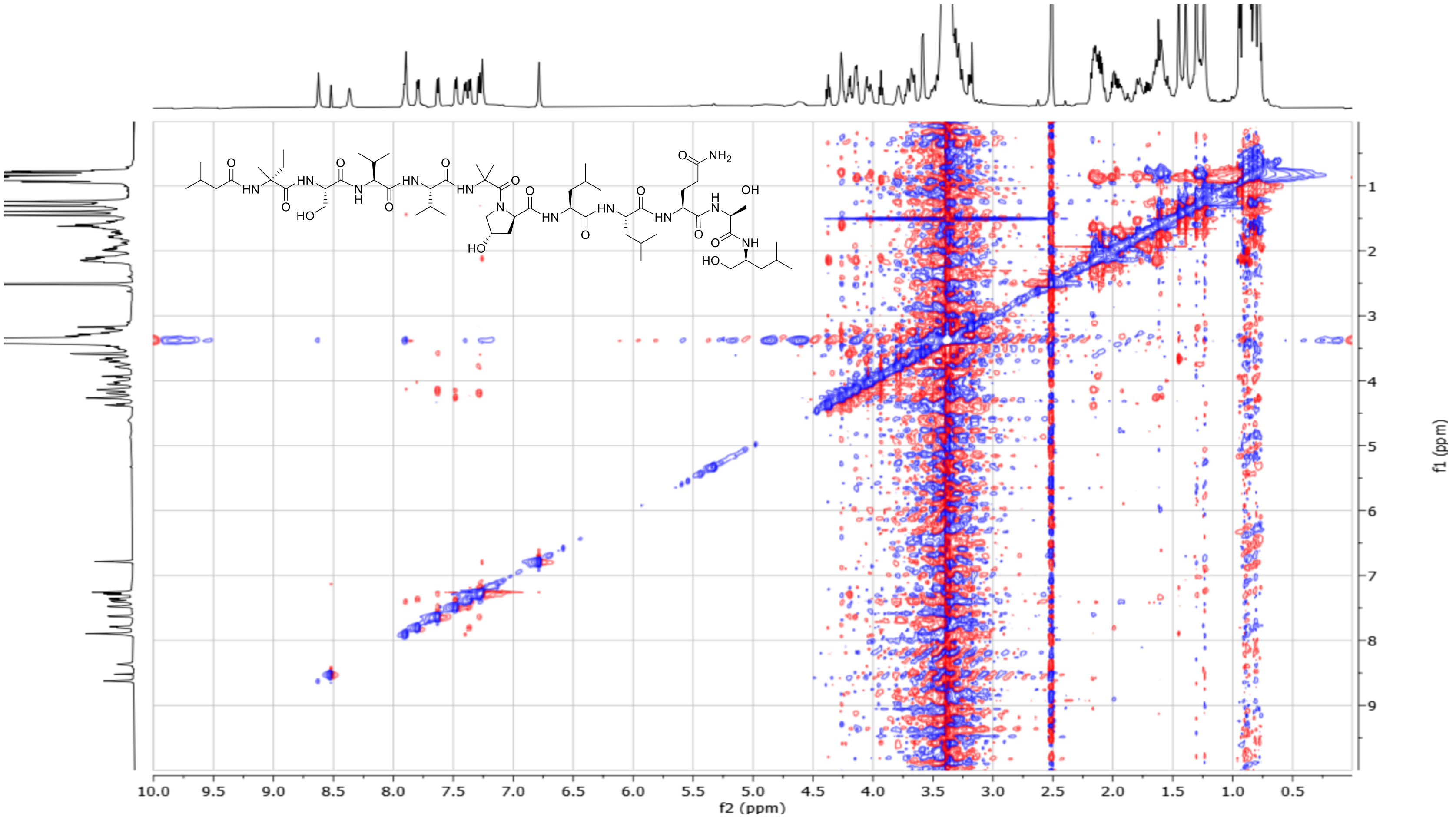

Figure S8. ROESY NMR (600 MHz, DMSO-d_6) spectrum of **1**

Analysis Report

Agilent | Trusted Answers

Sample Information

**Name**	176B170C	**Data File Path**	C:\Users\bokeschh\Desktop\020620\176B170C.d
**Sample ID**		**Acq. Time (Local)**	2/6/2020 10:45:02 AM (UTC-05:00)
**Instrument**	Instrument 1	**Method Path (Acq)**	C:\Users\admin\Desktop\methods\FIA_SM_LowFlow.m
**MS Type**	QTOF	**Version (Acq SW)**	6200 series TOF/6500 series Q-TOF B.09.00 (B9044.1 SP1)
**Inj. Vol. (ul)**	1	**IRM Status**	Success
**Position**	Vial 1	**Method Path (DA)**	
**Plate Pos.**		**Target Source Path**	
**Operator**		**Result Summary**	

Sample Chromatograms

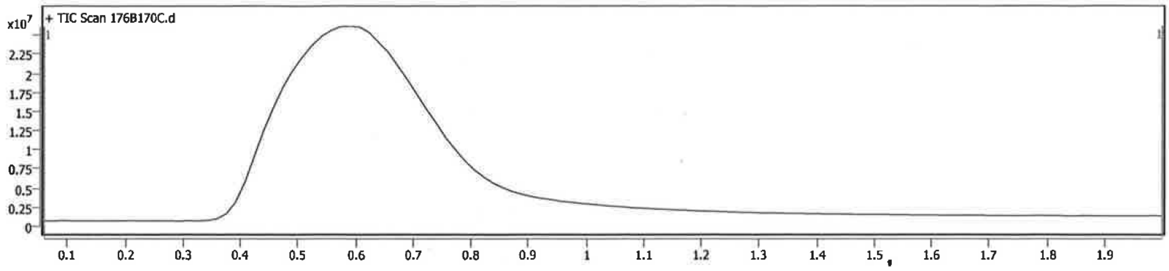

Sample Spectra

+ Scan (rt: 0.375-0.855 min) **C58 H104 N12 O16**

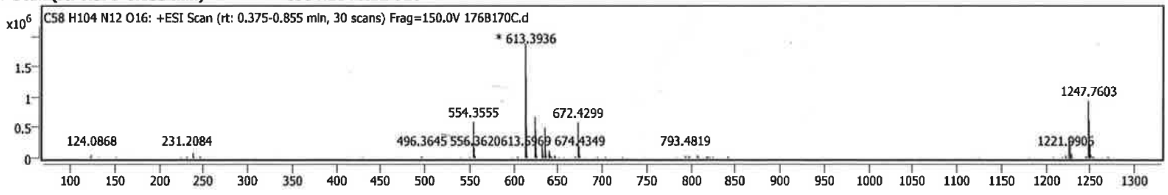

Spectrum Identification Table

Best ID Source	Name	Formula	Species	m/z	Diff (ppm)	CAS	Score	Score (Lib)	Score (DB)	Score (MFG)	Lib/DB
Yes MFG		C58 H104 N12 O16	(M+H)+	1225.7773	0.29		99.30			99.30	

MassHunter Qual 10.0
(End of Report)

Figure S9. HRESI-MS data for **1**

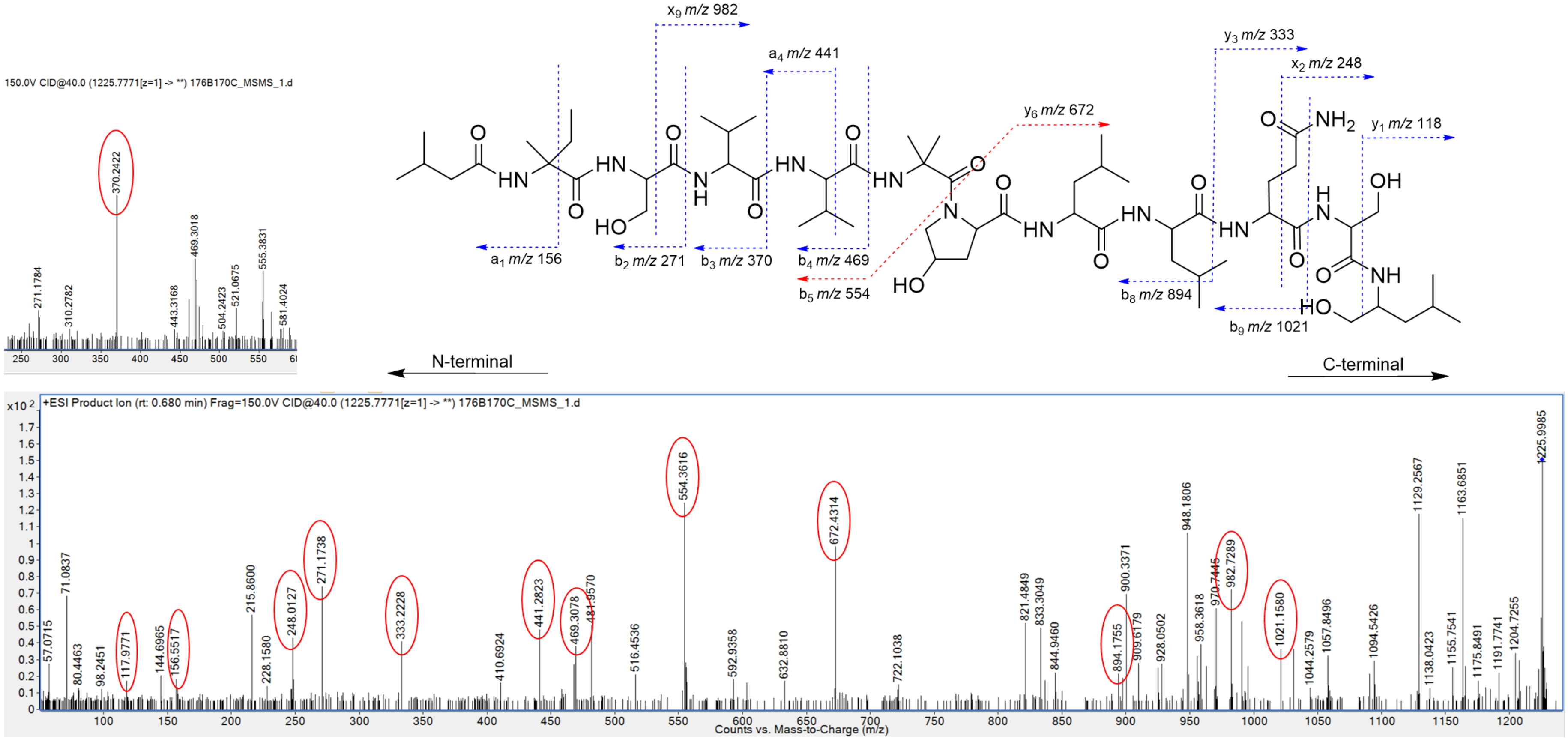

Figure S10. HR ESI-MS/MS fragmentations for **1**

Advanced Marfey's analysis of **1**

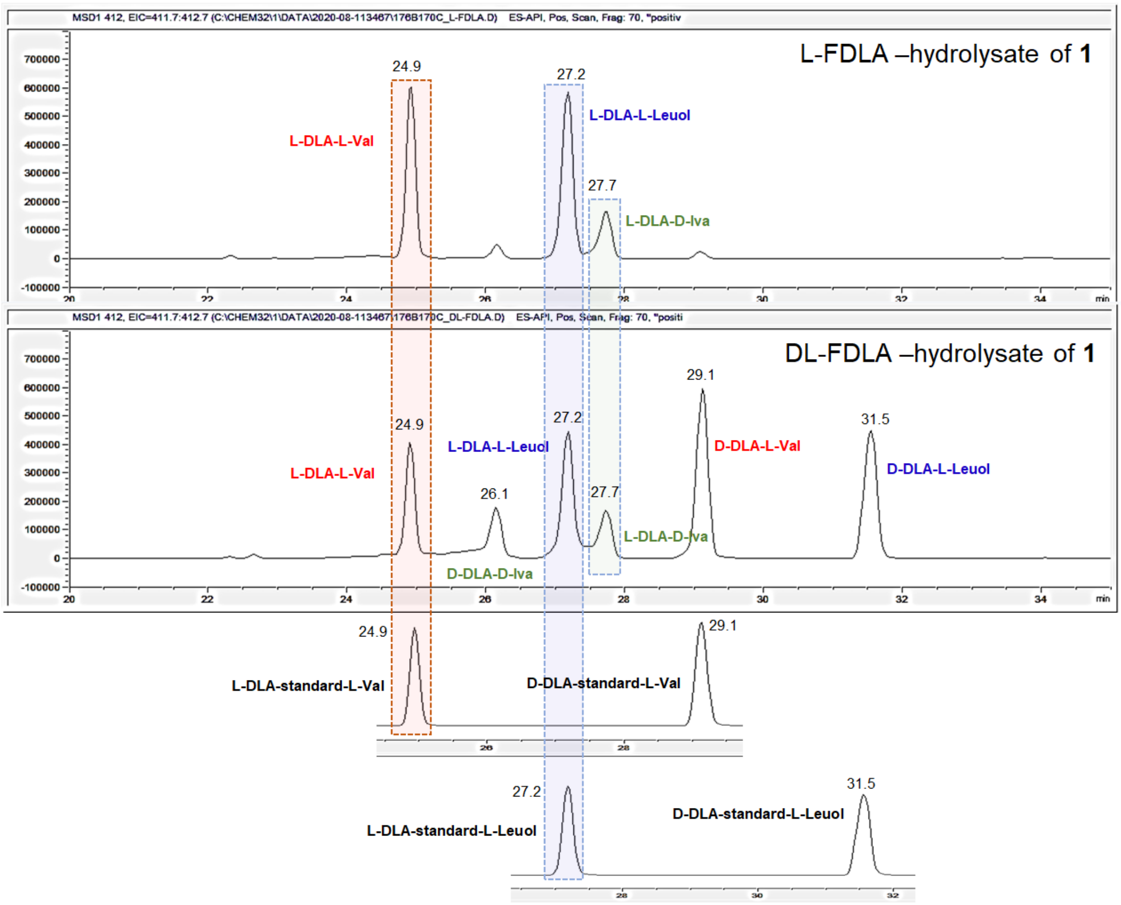

Figure S11. Ion chromatograms of the L-FDLA derivatives (top) and the D/L-FDLA derivatives (middle) of the hydrolysis product of **1 and** the D/L-FDLA derivatives of the standard L-Val and L-Leuol (bottom) in positive ion mode monitoring at *m/z* 412 for Iva, Val, and Leuol residues.

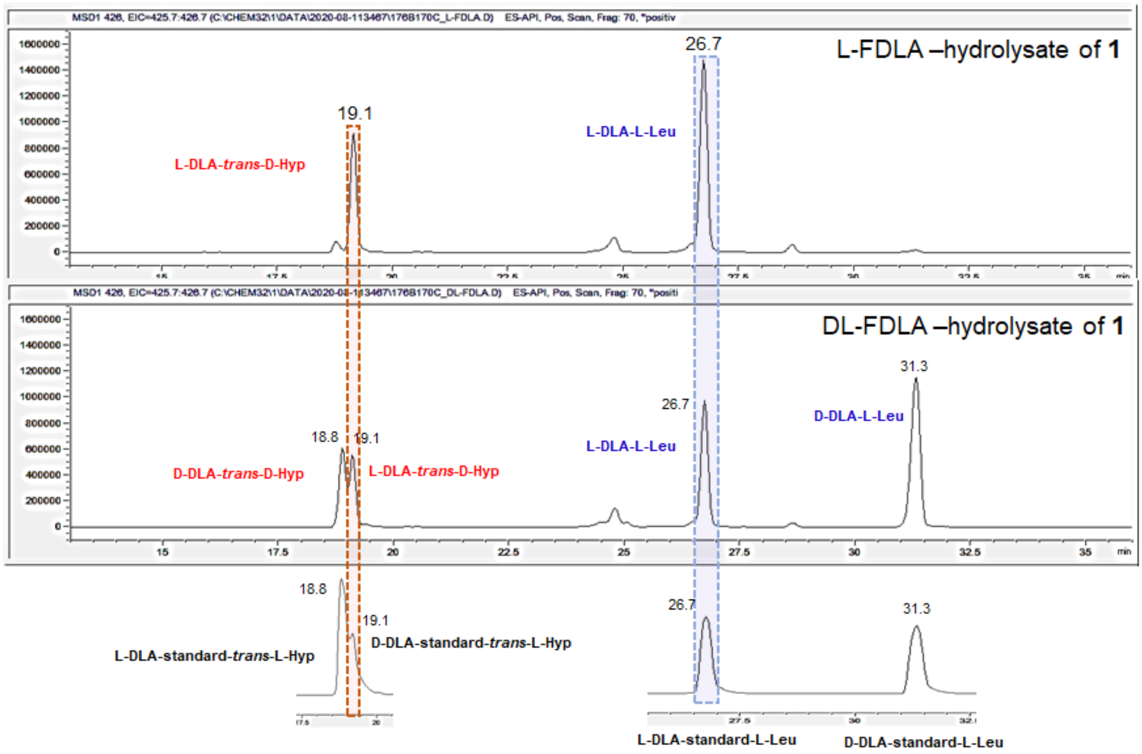

Figure S12. Ion chromatograms of the L-FDLA derivatives (top) and the D/L-FDLA derivatives (middle) of the hydrolysis product of **1 and** the D/L-FDLA derivatives of the standard *trans*-L-Hyp and L-Leu (bottom) in positive ion mode monitoring at *m/z* 426 for *trans*-Hyp and Leu residues.

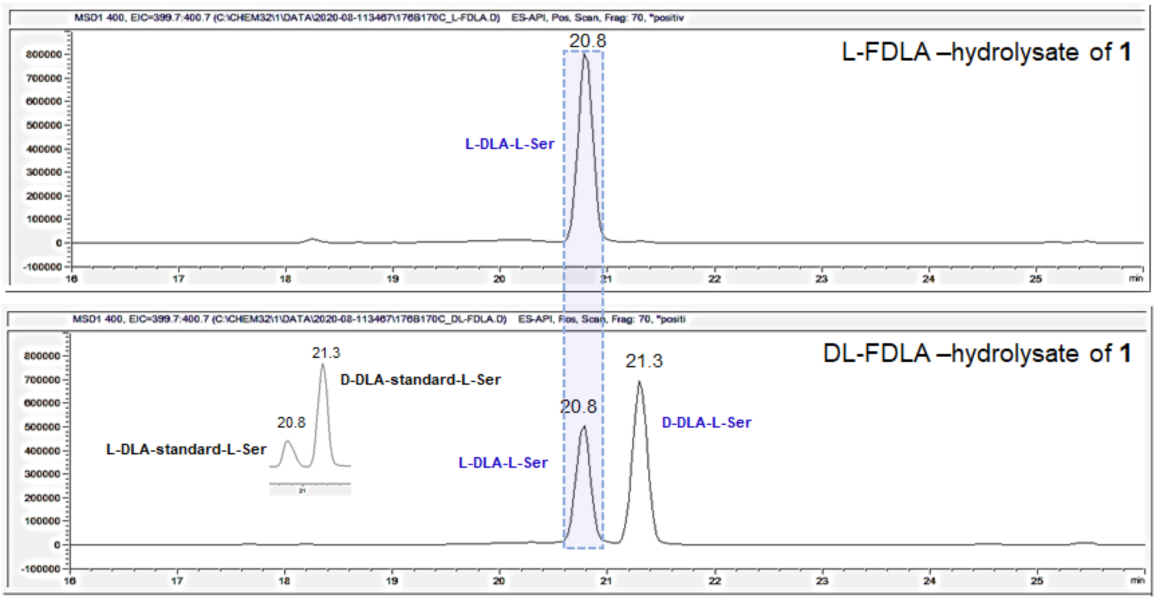

Figure S13. Ion chromatograms of the L-FDLA derivatives (top) and the D/L-FDLA derivatives (bottom) of the hydrolysis product of **1 and** the D/L-FDLA derivatives of the standard L-Ser in positive ion mode monitoring at *m/z* 400 for Ser residue.

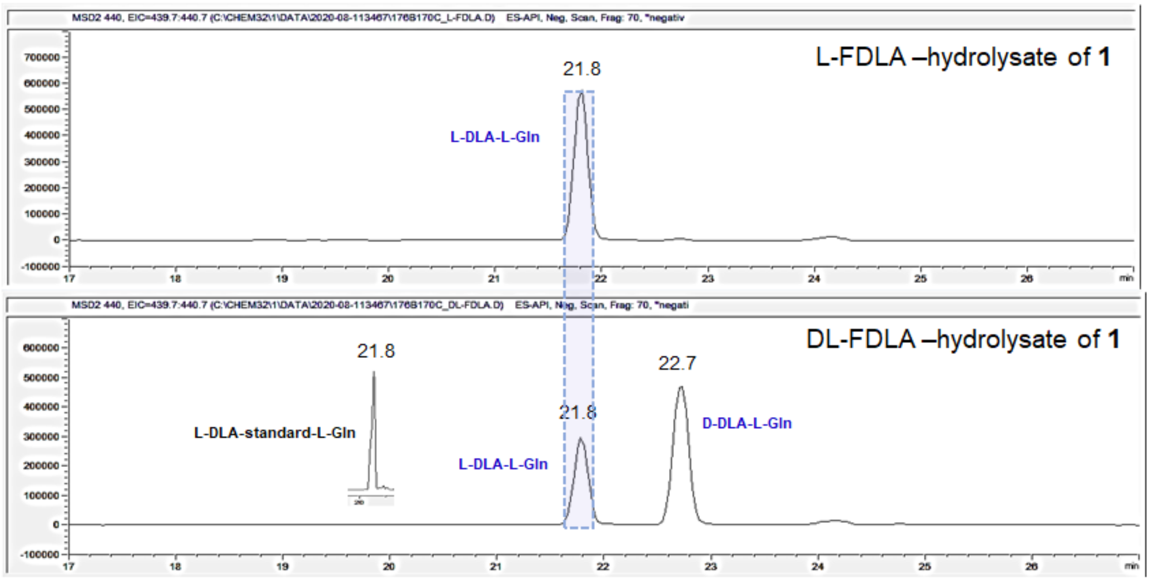

Figure S14. Ion chromatograms of the L-FDLA derivatives (top) and the D/L-FDLA derivatives (bottom) of the hydrolysis product of **1 and** the D/L-FDLA derivatives of the standard L-Gln in negative ion mode monitoring at *m/z* 440 for Gln residue.

Figure S15. ECD spectrum of roseabol A (**1**)

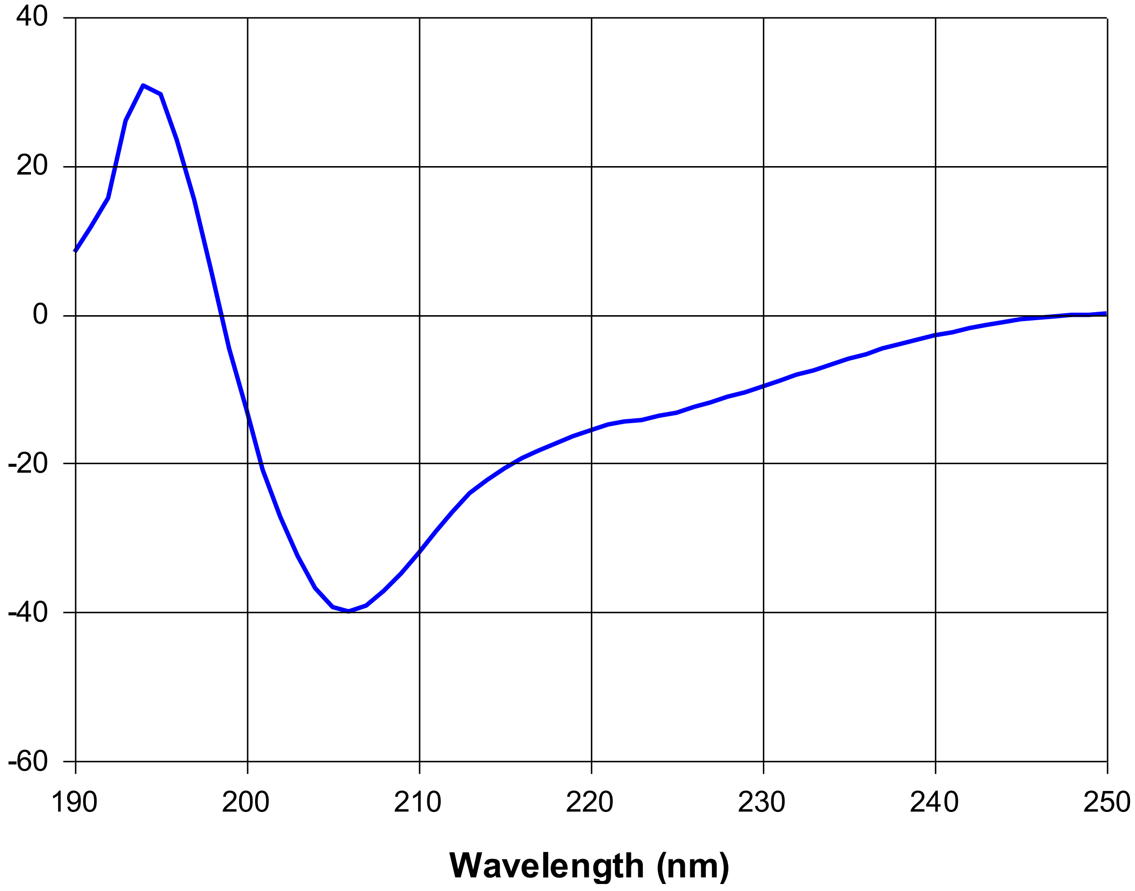

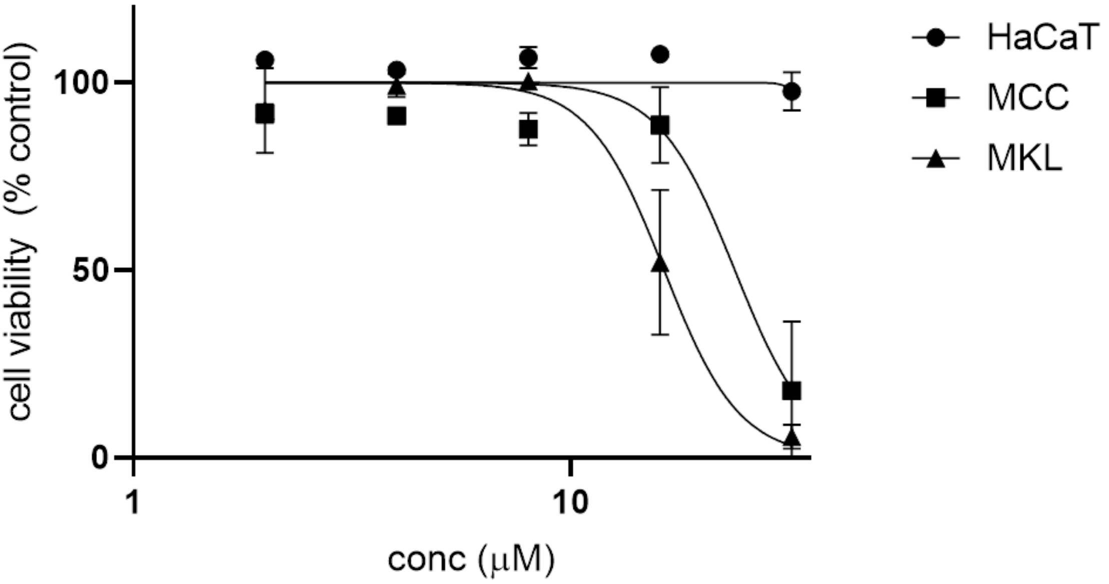

Figure S16. Merkel cell carcinoma cytotoxic activity for compound **2** assessed against the MKL-1 (solid triangles) and MCC26 (open circles) cell lines, with HaCaT cells (solid circles) as a noncancerous control. Bortezomib was the positive control with an IC_{50} of 13.1 +/- 5.8 nM for the three cell lines. Samples were tested in duplicate and the compound purity was assessed as > 90% by HPLC, NMR, and MS analysis.
